# Predictors of Participation Restriction in Midlife Caregivers: An Exploratory Study

**DOI:** 10.1093/geroni/igab046.1532

**Published:** 2021-12-17

**Authors:** Tara Klinedinst, Scott Beach, Heidi Donovan, Grace Campbell

**Affiliations:** 1 University of Pittsburgh, Pittsburgh, Pennsylvania, United States; 2 University of Pittsburgh, University of Pittsburgh, Pennsylvania, United States

## Abstract

Mid-life family caregivers (CGs) are at risk for participation restrictions (reduced engagement in valued roles and activities) due to competing demands of work, parenting, and family caregiving responsibilities. When CGs experience participation restrictions, quality of care for care recipients (CR) decreases, yet CG burden and risk for poor health increases. The purpose of this study was to identify the factors contributing to decreased participation in mid-life CGs. Participants were CGs aged 45-64 years (n = 677) from the National Study of Caregiving/National Health and Aging Trends Study. We used multivariate logistic regression to determine attributes of CGs, CRs, and the care situation that independently contribute to participation restrictions. We found that negative aspects of caregiving (OR = 1.51, 95% CI = 1.33, 1.71) and CR depression and anxiety (OR = 0.90, 95% CI = 0.83, 0.99) significantly predicted participation restrictions (p < 0.05). Positive aspects of care (OR = 0.87, 95% CI = 0.74, 1.01), frequency of helping with chores (OR = 1.30, 95% CI = 0.98, 1.70), frequency of providing personal care (OR = 1.24, 95% CI = 0.97, 1.59), and frequency of providing help getting around the home (OR = 1.30, 95% CI = 0.97, 1.75) showed trends for association with participation restrictions (p < 0.10). We identified factors that are related to participation restriction in mid-life CGs. Some of these factors (e.g., positive and negative aspects of caregiving, frequency of assistance provided) are potentially modifiable intervention targets that could bolster participation in this at-risk group.

